# Effects and Mechanisms of Taohong Siwu Decoction on the Prevention and Treatment of Myocardial Injury

**DOI:** 10.3389/fphar.2022.816347

**Published:** 2022-01-26

**Authors:** Chang-Le Shao, Guo-Hong Cui, Hai-Dong Guo

**Affiliations:** ^1^ Academy of Integrative Medicine, Shanghai University of Traditional Chinese Medicine, Shanghai, China; ^2^ Department of Anatomy, School of Basic Medicine, Shanghai University of Traditional Chinese Medicine, Shanghai, China; ^3^ Department of Neurology, Shanghai No. 9 People’s Hospital, Shanghai Jiaotong University School of Medicine, Shanghai, China

**Keywords:** taohong siwu decoction, cardiovascular diseases, myocardial injury, cardiomyocytes protection, mechanisms and applications

## Abstract

Taohong Siwu decoction (THSWD) is one of the classic prescriptions for promoting blood circulation and removing blood stasis. With the continuous in-depth excavation in basic and clinical research, it has been found that THSWD has made greater progress in the prevention and treatment of cardiovascular diseases. Mechanisms of the current studies have shown that it could prevent and treat the myocardial injury by inhibiting inflammatory reaction, antioxidant stress, inhibiting platelet aggregation, prolonging clotting time, anti-fibrosis, reducing blood lipids, anti-atherosclerosis, improving hemorheology and vascular pathological changes, regulating related signal pathways and other mechanisms to prevent and treat the myocardial injury, so as to protect cardiomyocytes and improve cardiac function. Many clinical studies have shown that THSWD is effective in the prevention and treatment of cardiovascular diseases related to myocardial injuries, such as coronary heart disease angina pectoris (CHD-AP), and myocardial infarction. In clinical practice, it is often used by adding and subtracting prescriptions, the combination of compound prescriptions and combinations of chemicals and so on. However, there are some limitations and uncertainties in both basic and clinical research of prescriptions. According to the current research, although the molecular biological mechanism of various active ingredients needs to be further clarified, and the composition and dose of the drug have not been standardized and quantified, this study still has exploration for scientific research and clinical practice. Therefore, this review mainly discusses the basic mechanisms and clinical applications of THSWD in the prevention and treatment of the myocardial injury caused by CHD-AP and myocardial infarction. The authors hope to provide valuable ideas and references for researchers and clinicians.

## Introduction

Due to the change of people’s lifestyle and the improvement of living standards, the incidence of metabolic diseases, cardiovascular diseases and many other diseases is gradually increasing and showing a trend of youthfulness. Among them, cardiovascular diseases is the number one killer endangering human life and health (Chen and Gao, 2016; Benjamin et al., 2018). Myocardial injury (MI) can be caused by a variety of causes, resulting in the occurrence and development of many diseases, among which coronary heart disease angina pectoris (CHD-AP) and myocardial ischemia-reperfusion injury (MIRI) are the most common cardiovascular diseases leading to MI in clinical practice.

A series of traumatic changes such as myocardial ultrastructure, energy metabolism, cardiac function and electrophysiology caused by MIRI during ischemia are more prominent after vascular recanalization (Kalogeris et al., 2012; Kalogeris et al., 2016), and severe arrhythmias can lead to sudden death. At present, it is considered that the mechanisms of MIRI are mainly related to the production of a large number of oxygen free radicals, calcium overload, leukocyte inflammation and the lack of high-energy phosphate compounds (Sanada et al., 2011; Raedschelders et al., 2012; Jennings, 2013; Xu, 2014; Wang and Zhang, 2018; Xia and Dong, 2019; Gao et al., 2020). Coronary heart disease (CHD, coronary atherosclerotic heart disease) is a heart disease caused by myocardial ischemia, hypoxia and even necrosis (Kachur et al., 2017). CHD is a common and frequently-occurring disease among the middle and old aged people. However, its incidence is getting younger in recent years, which seriously threatens to human health and life. With the development of atherosclerotic plaque of CHD, the coronary artery will be blocked gradually, resulting in decline of cardiac functions, adaptive changes and even damage of cardiomyocytes. Moreover, stable angina pectoris (SAP) is easy to develop into unstable angina pectoris (UAP) and acute myocardial infarction clinically (Tegn et al., 2016).

Chinese medicine (CM) is a valuable natural resource in China’s drug inventory. Its multi-component, multi-pathway and multi-target characteristics have shown unique advantages in the prevention and treatment of MI (Li et al., 2021). Although the chemical composition of CM is very complex (there may be dozens or even hundreds of kinds), their characteristic is the material basis of its effect on the prevention and treatment of diseases (Zhang and Li, 2015). Under the guidance of the holistic concept of Traditional Chinese Medicine (TCM), the comprehensive pharmacological effects of multi-pathways and multi-targets shown by the effective components of CM (Jiang and Gao, 2018), correspond to the occurrence mechanism of the disease to a certain extent. CM can improve multiple links of myocardial ischemic injury and reperfusion injury at the same time, as well as protect undamaged tissues, which is the greatest advantage of TCM in the prevention and treatment of cardiovascular disease. In addition, CM and its compound prescription also have the characteristics of outstanding curative effect, small side effect, good economy and low cost, which provides the possibility for the popularization, standardization and internationalization of TCM. This review mainly discusses the basic mechanisms and clinical applications of Taohong Siwu decoction in the prevention and treatment of MI caused by CHD-AP and myocardial infarction, hoping to provide valuable ideas and references for researchers and clinicians.

## Taohong Siwu Decoction

**FIGURE 1:**
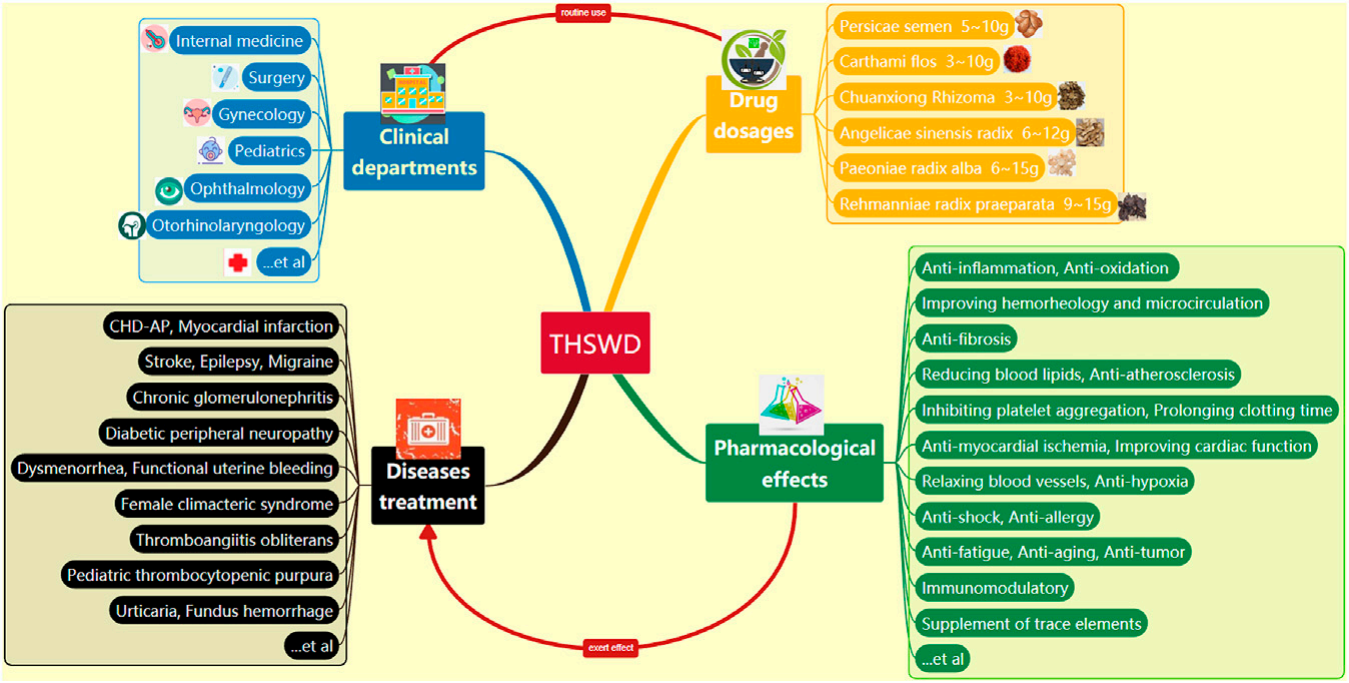
Clinical applications and pharmacological effects of THSWD (THSWD: Tao Hong Siwu decoction).

Taohong Siwu decoction (THSWD) was initially used exclusively as a basic prescription for gynecological menstruation regulation, then gradually expanded to other clinical treatments (Figure 1). Now it is widely used in internal medicine, surgery, gynecology, pediatrics, ophthalmology, otorhinolaryngology, and other clinical departments (Nie and Cheng, 2020). It can prevent and treat CHD-AP, myocardial infarction, stroke, migraine, epilepsy, chronic glomerulonephritis, diabetic peripheral neuropathy, functional uterine bleeding, dysmenorrhea, female climacteric syndrome, thromboangiitis obliterans, pediatric thrombocytopenic purpura, urticaria, fundus hemorrhage and other cardiovascular diseases, cerebral and renal vascular, blood and neurological diseases (Lian and Qin, 2010; Zhang and Peng, 2011; Zhang, 2014a; Deng, 2021).

THSWD, also known as Jiawei Siwu decoction, as one of the classic prescriptions for promoting blood circulation and nourishing blood, comes from the Heart Tips of Yizong Jinjian Gynecology (Volume 44) written by Wu Qian (Qing Dynasty) (Qian, 1980). The original article states that “if there are many lumps of blood, purple and sticky, there is blood stasis inside, with Siwu decoction (*Chuanxiong Rhizoma, Angelicae sinensis radix, Paeoniae radix alba, Rehmanniae radix praeparata*) plus *Persicae semen, Carthami flos* broken, which is called Taohong Siwu Decoction.” Modern pharmacological studies have shown that many active ingredients in THSWD have the effects of anti-inflammation (Mao, 2016; Liu, 2019a), improving hemorheology and microcirculation (Xie and Luo, 2008; Yi and Peng, 2011), reducing blood lipids, anti-atherosclerosis (Zhou, 2003; Xie and Luo, 2008), anti-myocardial ischemia, improving cardiac function, relaxing blood vessels, and inhibiting platelet aggregation (Han et al., 2010; Han and Peng, 2010), prolonging clotting time, anti-fibrosis (Tan et al., 2021), anti-hypoxia, anti-oxidation (Luo et al., 2019), anti-aging, anti-tumor, immunomodulatory, anti-fatigue (Li et al., 2012), anti-shock, anti-allergy, and supplement of trace elements (Wu, 2011; Zhang and Wang, 2011; Li and Guo, 2016; Wang, 2021; Xiang and Shi, 2021). The general clinical dosage of THSWD is 5–10 g *Persicae semen*, 3–10 g *Carthami flos*, 3–10 g *Chuanxiong Rhizoma*, 6–12 g *Angelicae sinensis radix*, 6–15 g *Paeoniae radix alba*, and 9–15 g *Rehmanniae radix praeparata* (Chinese Pharmacopoeia Commission, 2015; Ji and Lian, 2016; Gao and Zhang, 2017), but the specific dosage of medicinal ingredients needs to be determined according to specific symptoms of the disease (Figure 1).

## Mechanisms of Taohong Siwu Decoction in Prevention and Treatment of MI

**FIGURE 2:**
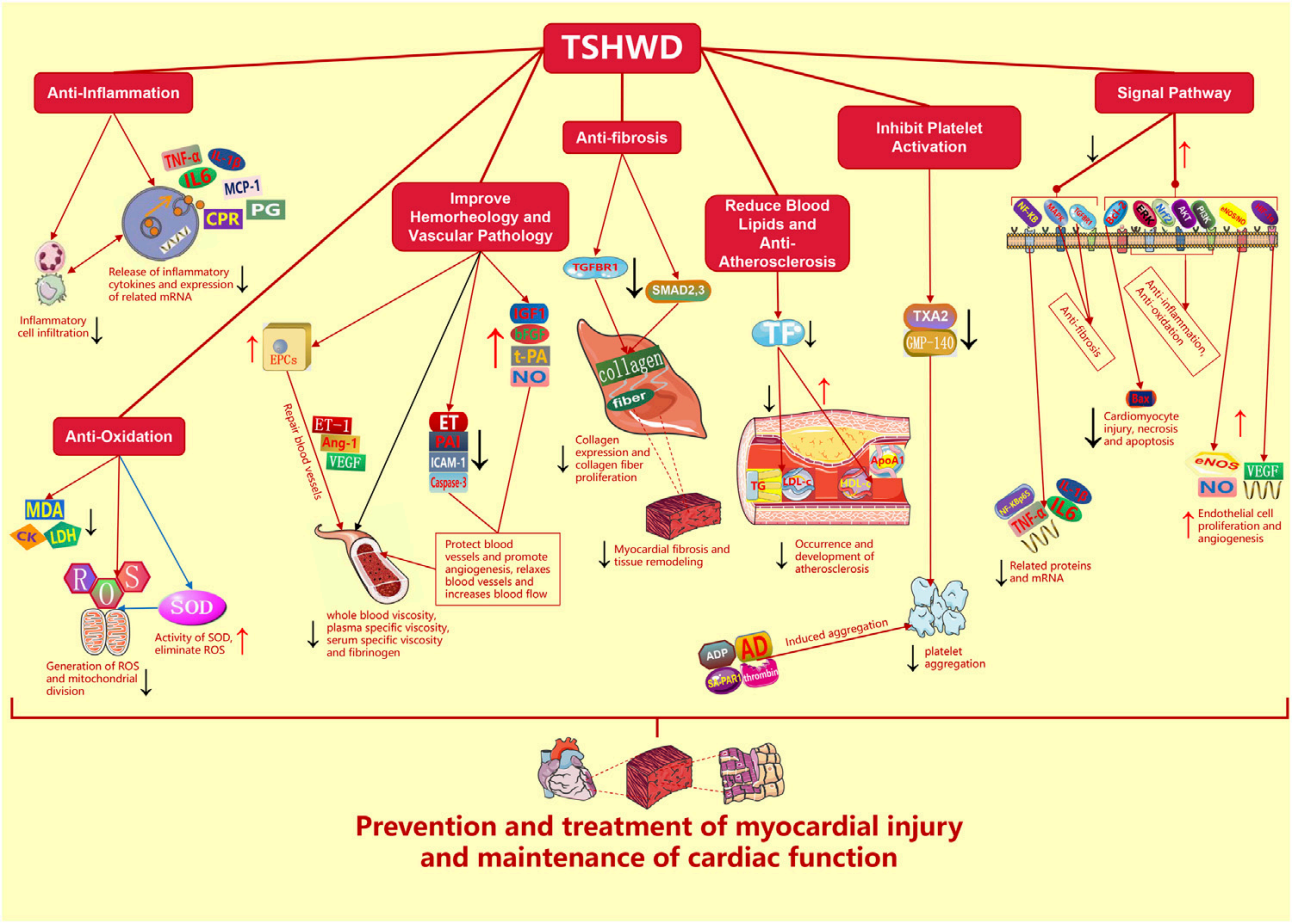
Mechanisms of THSWD in prevention and treatment of MI (THSWD: Taohong Siwu decoction, MI: myocardial injury, TNF-α: tumor necrosis factor-α, IL-1β: interleukin-1β, IL-6: interleukin-6, IL-8: interleukin-8, ROS: reactive oxygen species, MCP-1: monocyte chemoattractant protein-1, LPS: lipopolysaccharide, NF-κB: nuclear factor-κβ, hs-CRP: hypersensitive C-reactive protein, SOD: superoxide dismutase, PG: prostaglandins, LDL: low density lipoprotein, CK: creatine kinase, LDH: lactate dehydrogenase, MDA: malondialdehyde, bFGF: basic fibroblast growth factor, IGF-1: insulin-like growth factor-1, EPCs: endothelial progenitor cells, ET-1: endothelin-1, Ang-1: angiopoietin-1, VEGF: vascular endothelial growth factor, PAI: plasminogen activator inhibitor, ICAM-1: intercellular adhesion molecule-1, t-PA: tissue plasminogen activator, Caspase-3: cysteine aspartate protease-3, TGFBR1: transforming growth factor 1 receptor, SMAD2, 3: the phosphorylation of signal transduction protein 2,3, TC: total cholesterol, TG: triglyceride, LDL-c: low density lipoprotein-C, HDL-c: high density lipoprotein-C, TF: tissue factor, ApoA1: apolipoprotein A1, PLT: platelets, ADP: adenosine diphosphate, AD: adrenaline, TXA2: thromboxane A2, GMP-140: granule membrane protein-140, SA-PAR1: selective agonists of protease-activated receptor-1, HIF-1α: hypoxia inducible factor-1α, eNOS/NO: endothelial nitric oxide synthase/nitric oxide, Bcl-2: B lymphocyte tumor-2, Bax: Bcl-2-associated X protein, Nrf2: NF-E2 related factor 2, PI3K: phosphatidylinositol 3-kinase, ERK: extracellular regulated protein kinases, MAPK: mitogen-activated protein kinase).

The mechanisms of THSWD in prevention and treatment of MI inluding anti-inflammation, anti-oxidation, improving hemorheology and vascular pathology, anti-fibrosis, reducing blood lipids and anti-atherosclerosis, inhibiting platelet aggregation and prolonging clotting time, and regulting some signal pathways (Figure 2).

### Anti-Inflammation

Excessive inflammatory reaction is harmful to the human body. The inflammatory reaction is caused by the action of inflammatory factors on the body, resulting in local tissue and cell damage, even degeneration and necrosis. Among the many inflammatory cytokines, tumor necrosis factor-α (TNF-α), interleukin-1β (IL-1β), interleukin-6 (IL-6), interleukin-8 (IL-8) and so on play a major role in MI. THSWD can down-regulate the levels of serum inflammatory factors such as IL-6 and TNF-α (Liu, 2019a). The main mechanisms may be to inhibit the uncontrolled release of TNF-α and IL-6, remove inflammatory mediators, reduce inflammatory exudation, promote inflammatory absorption, thus inhibiting the progress of inflammatory reaction. The experiment showed that the serum-containing THSWD could significantly inhibit the increase of the content of reactive oxygen species (ROS) and the mRNA expression of TNF-α, IL-1β and monocyte chemoattractant protein-1 (MCP-1) induced by lipopolysaccharide (LPS) (Zhang, 2014b). Paeoniflorin, an intrinsic component of *Paeoniae radix alba*, can not only inhibit the expression of the above mRNA, but also reduce the LPS induced neutrophil/leukocyte infiltration (Zhou et al., 2013; Chen et al., 2015; Zhai and Guo, 2016). In addition, Kaempferol is a main component of *Carthami flos*, which could ameliorate inflammatory response in hyperglycemia-induced cardiac injury (Chen et al., 2018a). Oral adminstration of *Carthami flos* could induce macrophage activation (Choi et al., 2007), and baicalin, an ingredient of *Carthami flos*, could regulate macrophages polarization, thereby alleviating MIRI and inflammation (Xu et al., 2020).

Besides, inflammatory reaction significantly affects the formation and development of atherosclerosis (AS) plaques, and determines the formation speed and stability of plaques (Conti and Shaik-Dasthagirisaeb, 2015). Serum hypersensitive C-reactive protein (hs-CRP) is the most commonly used inflammatory marker in the clinical (Yayan, 2013). CRP can not only reflect the occurrence and development of AS, but also damage vascular endothelial cells through direct infiltration or indirect production of cytokines, and activate complements to aggravate myocardial injury (TIu et al., 2013; Zang and He, 2015). The study showed that the serum hs-CRP of the observation group decreased significantly after treatment with THSWD (Chu and Sun, 2014; Haoli, 2018; Lujuan and Wang, 2018), indicating that the inflammatory reaction was alleviated, but its mechanism is not clear. Furthermore, THSWD can enhance the activity of superoxide dismutase (SOD) (Luo and Zhou, 2014), inhibit the synthesis and release of prostaglandins (PG) and other inflammatory factors, thus improving inflammatory response, and preventing and repairing cell and tissue damage.

### Anti-Oxidation

Oxidative stress refers to the state of imbalance between oxidation and anti-oxidation *in vivo*, which tends to oxidation, resulting in inflammatory infiltration of neutrophils, increased secretion of protease, and the production of a large number of oxidation intermediates. Oxidative stress is a negative effect produced by free radicals in the body, and is considered to be an important factor in aging and diseases. Reactive oxygen species (ROS), including free radicals and non-free radical oxygen intermediates, play a key role in vascular endothelial dysfunction, low-density lipoprotein (LDL) oxidation, and inflammatory events in the initiation and development of atherosclerotic lesions (Negre-Salvayre et al., 2020).

Compared with the control group, the THSWD group significantly reduced the division of mitochondria and the production of mitochondrial ROS (Luo et al., 2019), and both its ethanol extract and water extract had a scavenging effect on 1,1-diphenyl-2-picrylhydrazyl radical (Yang et al., 2011), thus inhibiting the oxidative stress reaction and reducing the damage of myocardial cells (Liu et al., 2011; Yu and Hong, 2016). THSWD can decrease the levels of serum creatine kinase (CK) and lactate dehydrogenase (LDH) in myocardial tissue during acute myocardial ischemia (Zhu and Zhang, 2003; Luo and Zhou, 2014), thus reducing myocardial cell necrosis. Meanwhile, its effective component paeoniflorin can reduce the content of malondialdehyde (MDA) and enhance the activity of SOD (Zhang and Zhu, 2003; Liu, 2018; Wu et al., 2020a; He and Wang, 2020). SOD has a strong antioxidant capacity, can quickly decompose excessive oxygen free radicals in the human body, eliminate lipid peroxidation in tissues and cells, and then prevent the injury of cardiomyocytes.

Furthermore, the study reveals that kaempferol could protect the mouse heart and H9c2 cells from pathological oxidative stress via antioxidant activity (Zhou et al., 2015; Feng et al., 2017). Luteolin from *Carthami flos* could improve cardiac function, alleviate mitochondrial injury, decrease oxidative stress (Luo et al., 2017), inhibited cardiac apoptosis and enhanced autophagy (Wu et al., 2020b). Quercetin from *Carthami flos* also appeared to affect heart mitochondrial function (Ruiz et al., 2015), and could relieve cardiac oxidative stress, so as to exhibit cardioprotective effects (Roslan et al., 2017). β-Sitosterol is widely found in *Persicae semen*, *Carthami flos*, *Angelicae sinensis radix*, *Paeoniae radix alba*, *Rehmanniae radix praeparata*. β-Sitosterol pretreatment could cause an increase in superoxide dismutase and glutathione activities and a decrease in malondialdehyde levels in the heart (Koc et al., 2021), indicating its cardioprotective effects were related to anti-oxidative stress. Moreover, β-Sitosterol produced an up-regulation of cellular glutathione redox cycling and protected against hypoxia/reoxygenation-induced apoptosis in H9c2 cells (Wong et al., 2014).

### Improve Hemorheology and Vascular Pathology

Hemorheology includes the rheology of blood vessels, the fluidity, viscosity, deformability and coagulability of blood. THSWD can decrease the whole blood viscosity (Xie and Luo, 2008; Liu et al., 2014; Luo and Zhou, 2014; Zhou and Shu, 2017; Wang and Wang, 2018; Jinxia et al., 2021), plasma specific viscosity, serum specific viscosity and fibrinogen (Han and Xu, 2007; Lujuan and Wang, 2018), and significantly increase the expression of basic fibroblast growth factor (bFGF) and insulin-like growth factor-1 (IGF-1) (Luo et al., 2019). These cytokines can promote neovascularization and protect the activity of cardiomyocytes. Studies have shown that the decrease of the above-mentioned indexes may be due to the pharmacological effect of *Carthamus tinctorius* lutein (Li et al., 2009). Moreover, *Chuanxiong Rhizoma* and *Angelicae sinensis radix* had evident angiogenic effects by promoting the endothelial cell proliferation and stimulating quantity of vessels (Meng et al., 2008). The changes in hemorheology and the formation of neovascularization improve the microcirculation of the heart and the microenvironment of cardiomyocytes, which provide potential possibilities for the prevention and treatment of cardiomyocyte injury.

THSWD promotes the expression of IGF-1 (Luo et al., 2019). IGF-1 can dilate blood vessels, reduces vascular resistance and increase blood flow to the heart, thus protecting cardiomyocytes and improving cardiac function. In addition, the left ventricular end-systolic volume of the model rats was significantly decreased after 4 weeks of THSWD treatment, which increased the cardiac ejection fraction, and improved the left ventricular short-axis shortening rate and left ventricular systolic function (Zhu and Zhang, 2003; Luo et al., 2019). Its internal mechanism may be that paeoniflorin, one of its active components, alleviates the decline of cardiac function caused by myocardial ischemia (Zhai and Guo, 2016), and studies have shown that paeoniflorin can significantly attenuates chamber dilatation and dysfunction of left ventricle caused by pressure overload (Zhou et al., 2013).

In terms of vascular protection, since endothelial progenitor cells (EPCs) can repair the injury of vascular endothelium (Hunting et al., 2005; Briasoulis et al., 2011; Toya and Malik, 2012; Zhang et al., 2014; Hu et al., 2019; Leal et al., 2019), the researchers found that THSWD can reduce the damage of vascular endothelial cells and maintain the normal secretory function of blood vessels by improving the functional activity and increasing the number of EPCs (Li et al., 2014; Li et al., 2015; Wang and Jiang, 2019). The mechanism of vascular protection and repair may be related to regulation of endothelin-1 (ET-1), angiopoietin-1 (Ang-1), and vascular endothelial growth factor (VEGF) in serum (Wang and Han, 2017). It was found that the contents of ET with vasoconstriction, plasminogen activator inhibitor (PAI) and intercellular adhesion molecule-1 (ICAM-1) with fibrinolysis inhibition were decreased, while the contents of NO with vasodilation and tissue plasminogen activator (t-PA) with fibrinolysis promotion were increased after treating endothelial cells with serum of Modified THSWD (Wu et al., 2014). This suggests that Modified THSWD can obviously improve the dyssecretion of vascular endothelial cells caused by blood stasis and promote the migration of vascular endothelial cells, thus protecting the morphology and function of blood vessels. Furthermore, the serum containing THSWD can protect human umbilical vein endothelial cells from hydrogen peroxide damage and reduce the apoptosis rate of endothelial cells, and its mechanism of inhibiting apoptosis may be related to the decreased expression of cysteine aspartate protease-3 (Caspase-3) (Liu et al., 2013).

### Anti-Fibrosis

Fibrosis can occur in various tissues and organs, and its main pathological changes are the increase of fibrous connective tissue and the decrease of parenchymal cells. Continuous progress can lead to the destruction of organ structure, functional decline and even exhaustion, which seriously threatens human health and life. For the treatment of myocardial fibrosis after myocardial infarction, the latest researches have shown that THSWD can significantly reduce myocardial fibrosis and ensure stable cardiac function by inhibiting transforming growth factor 1 receptor (TGFBR1) (Yayan, 2013), reducing collagen deposition and inhibiting fibrosis proliferation (Luo et al., 2019). The underlying mechanism may be that paeoniflorin and other effective substances reduce the expression of collagen, inhibit the TGFBR1 signaling pathway and the phosphorylation of signal transduction protein 2,3 (SMAD2,3) (Zhou et al., 2013; Liu et al., 2019a; Tan et al., 2021). However, the overexpression of TGFBR1 can reduce or even reverse the anti-fibrosis effect of THSWD (Tan et al., 2021). In addition, the research has shown that THSWD can reduce myocardial interstitial collagen remodeling by inhibiting myocardial interstitial collagen fiber proliferation and collagen expression after myocardial infarction, and reducing the ratio of myocardial Ⅰ/Ⅲ collagen in a non-infarcted area (Zhou and Liu, 2011). Moreover, paeoniflorin not only could improve ventricular remodeling *via* inhibiting BNP, IL-6, TNF-α and increasing IL-10 levels (Chen et al., 2018b), but also could attenuate cardiac hypertrophy and cardiac fibrosis (Liu et al., 2019b).

### Reduce Blood Lipids and Anti-Atherosclerosis

The basic pathological process of CHD is that coronary artery fixed stenosis or increased microvascular resistance leads to decreased coronary blood flow, unstable atherosclerotic plaque rupture, erosion or bleeding, secondary platelet aggregation or thrombosis, resulting in a sharp increase in the degree of coronary artery stenosis or closure, and/or coronary artery functional changes (such as spasm), causing in myocardial ischemia and hypoxia injury. However, the increase of serum lipid level is one of the independent risk factors of CHD (Bragg and Walling, 2015). AS is the appearance of yellow substances such as cholesterol and lipids in the intima of large and middle arteries, which is mostly caused by fat metabolism disorders and neurovascular dysfunctions, and often leads to thrombosis, blood supply disorders and so on. Studies have shown that THSWD plays a certain role in reducing total cholesterol (TC), triglyceride (TG), low-density lipoprotein-C (LDL-c), LDL-c/TC ratio, and increasing high-density lipoprotein-C (HDL-c), HDL-c/TC ratio (Chen and Wang, 2005; Xie and Luo, 2008; Luo and Zhou, 2014; Liu, 2015a; Haoli, 2018; Chen et al., 2019; Li and Yan, 2020). One of the mechanisms may be that peach kernel oil significantly downregulates the protein expression of tissue factor (TF) (Hao et al., 2019), thereby inhibiting the formation of atherosclerotic plaques. Moreover, THSWD could significantly decrease the ratio of serum TG/HDL-c and increase the content of serum apolipoprotein A1 (ApoA1) (Zhou, 2003). The decrease of TC, TG, LDL-c, and the increase of LDL-c, ApoA1 help to reduce blood lipids, thus inhibiting the occurrence and development of AS. In addition, baicalin could ameliorate atherosclerotic lesion progression via lipid modulation in ApoE−/− mice with high-cholesterol diet (Liao et al., 2014). A kind of active ingredients from *Chuanxiong rhizoma*, tetramethylpyrazine (Ligustrazine) also exhibited cardioprotective effects on atherosclerosis and MIRI (Guo et al., 2016).

### Inhibit Platelet Aggregation and Prolong Clotting Time

The main functions of platelets (PLT) are coagulation, hemostasis and repair of damaged blood vessels. Since the surface sugar coating of platelets can absorb plasma proteins and coagulation factor Ⅲ, platelet granules also contain substances related to coagulation. Platelet activation consists of three steps: adhesion, aggregation and release. Activated platelets form platelet thrombus, which can cause thrombotic diseases such as acute myocardial infarction and ischemic stroke.

Studies have shown that THSWD can effectively reduce platelet adhesion rate in rats in a dose-dependent manner, and can inhibit platelet aggregation induced by adenosine diphosphate (ADP) and adrenaline (AD), and its effect is similar to that of aspirin (Han et al., 2010). Under the action of ADP (Liu et al., 2016), AD and other inducers, platelets can release a series of active substances, such as thromboxane A2 (TXA2), platelet granule membrane protein-140 (GMP-140), β-thromboglobulin, and platelet factor-4 to promote platelet aggregation (Liu and Yin, 2014). However, THSWD can significantly decrease the levels of plasma TXA2 and GMP-140 in rats (Han et al., 2010), thereby inhibiting platelet aggregation. In addition, within the range of 0.5–2.5 mg/ml, THSWD could inhibit platelet aggregation induced by thrombin and selective agonists of protease-activated receptor-1 (SA-PAR1) in a dose-dependent manner (Yin and Yang, 2012). The research has shown that THSWD can prolong thrombosis time and clotting time in rats (Liu and Yin, 2014), in which the prothrombin time is prolonged with the increase of the dose of *Carthamus tinctorius* lutein (Yin and Yang, 2012), showing an obvious anticoagulant effect. To sum up, THSWD can play a certain role in the three key links of platelet activation, and play the role of anti-platelet and prolonging clotting time.

### Signal Pathway

It has been found that THSWD can inhibit inflammatory reaction by regulating the NF-kB signaling pathway, and its internal mechanism may be to inhibit the expression of NF-kBp65, TNF-α protein, and its mRNA in myocardial tissue, and reduce the contents of IL-1β and IL-6 in serum (Shen and Shi, 2019), thus effectively protecting the structure and function of the myocardium. Moreover, THSWD can protect human brain microvascular endothelial cells from ischemic injury, which may enhance the expression of VEGF and the ability of cell antioxidation through hypoxia-inducible factor-1α (HIF-1α) signal pathway (Zhaojie and Han, 2018). It can also promote endothelial cell proliferation by up-regulating endothelial nitric oxide synthase/nitric oxide (eNOS/NO) mediated signal pathway (Xiaoxia, 2010), since eNOS/NO is an important factor in promoting angiogenesis (Yasuda, 2008). Through activating Nrf2 mediated HIF-1α pathway, baicalin can protect cardiomyocytes from apoptosis induced by hypoxia (Yu et al., 2019). Besides, THSWD can increase the expression of B lymphocyte tumor-2 (Bcl-2) gene, decrease the expression of Bcl-2-associated X protein (Bax), alleviate cardiomyocyte injury, inhibit cardiomyocyte necrosis and apoptosis (Li and Wang, 2009), thus preventing the pathological changes of myocardial ischemia and maintain cardiac function.

In studies of the effective components of THSWD, ferulic acid can activate the signal pathways of transcription factor NF-E2 related factor 2 (Nrf2), phosphatidylinositol 3- kinase (PI3K) and extracellular regulated protein kinases (ERK) to play an antioxidant role, thus protecting vascular endothelial cells from oxidative damage (Ma et al., 2010). Cardioprotective potential of amygdalin from *Persicae semen* could inhibit cardiac hypertrophy, oxidative stress and inflammatory responses through modulation of Nrf2 and NF-κB activation (Kung et al., 2021). Paeoniflorin can also reduce the expression of related mRNA (Zhou et al., 2013; Chen et al., 2015), inhibit NF-kB signaling pathway and activate PI3K/Akt signaling pathway (Zhai and Guo, 2016), thereby exerting anti-inflammatory and anti-oxidation effects. Luteolin and quercetin could protect diabetic cardiomyopathy against inflammation and oxidative stress injury *via* NF-kB pathway inhibition (Patel et al., 2018; Li et al., 2019; Chen et al., 2020). Moreover, the findings demonstrated that cardioprotective effects of lactone component from Ligusticum chuanxiong were related to restoration of autophagic flux through the activation of PI3K/Akt/mTOR signaling pathway (Wang et al., 2018a), and ligustrazine from *Chuanxiong rhizoma could* exert cardio protection through multiple signaling pathways in MIRI (Zheng et al., 2018).

Paeoniforin could attenuate myocardial fibrosis and improve cardiac function in CHF rats by down-regulating the p38 MAPK signaling pathway (Liu et al., 2020). Hydroxysafflor yellow A (HSYA) in *Carthami flos* has a protective effect on vascular endothelial injury induced by hypoxia. It may be that HSYA can increase the level of NO under hypoxia, up-regulate the ratio of Bcl-2/bax, the expression of eNOS-mRNA and VEGF-mRNA and its protein, enhance the accumulation of HIF-1α protein and its transcriptional activity (Ji et al., 2008; Ji et al., 2009), thus improving the viability of vascular endothelial cells under hypoxia and promoting endothelial cell proliferation and angiogenesis through VEGF/VEGF receptor (Song et al., 2005).

## Clinical Applications of Taohong Siwu Decoction in Prevention and Treatment of MI

In the clinical prevention and treatment of myocardial injury related diseases, THSWD can decrease serum TC, increase coronary blood flow, reduce myocardial oxygen consumption, resist myocardial ischemia, inhibit platelet aggregation and enhance the activity of the fibrinolytic enzyme system (Yang, 2007). It can also effectively reduce blood viscosity and the level of serum inflammation, thus improving the clinical effect (Lujuan and Wang, 2018). In addition, THSWD can inhibit the level of serum ET-1 in patients with coronary spastic AP of qi stagnation and blood stasis type, and its improvement of AP may be related to the decrease of ET-1 level and the improvement of vascular endothelial function (Chen, 2013). However, the efficacy of THSWD will decrease with the increase of the degree of AP, and it may only have a certain curative effect on mild and moderate AP (Yang, 2007). Moreover, the current clinical practical applications are mostly carried out in the way of combination formulas and combined chemical drugs, and there are fewer examples of THSWD alone (Table 1).

**TABLE 1:** Clinical applications of THSWD in prevention and treatment of MI.

	Drugs/Prescriptions	Diseases	Pharmacological effects	References
Application of combined chemicals	Sodium ozagrel	UAP	Improve microcirculation and myocardial ischemia	Jing and Wang (2009), Renhua and Li (2017)
	Shuxuening			Wang and Wang (2008)
	Salvia miltiorrhiza injection			Yu (2012)
	Agkistrodon halys antithrombotic enzyme			Rui (2002)
	Metoprolol	AP of qi deficiency and blood stasis syndrome	Regulate of hemorheology, improve left ventricular systolic function	Wang et al. (2018b), Wang and Wang (2018), Wang and Jiang (2019)
	Diltiazem	Coronary spasm AP	Improve the level of blood lipids	Song et al. (2005)
	Low molecular heparin calcium	ACS	Prevention and treatment of myocardial infarction	Zhu et al. (2011)
	Rosuvastatin	AP	Reduce blood lipid, improve myocardial blood supply and heart function	Haoli (2018)
	Isosorbide mononitrate			Yan et al. (2012)
	Atorvastatin	CHD	Improve efficacy and reduce side effects	Wang (2018)
Use of combined prescriptions	Zhenwu decoction	CSHF	Increase LVEF, delay the ventricular remodeling	Joung et al. (2003); Xiao and Chen. (2015); Xiao and Gao. (2017)
	Shenfu decoction		Increase LVEF	Wang and Li, (2017)
	Baoyuan decoction	AP of qi deficiency and blood stasis type	Dilate coronary artery, improve microcirculation	Song. (2010), Li and Zhang. (2018), Zhang. (2019)
	Gualou Xiebai banxia decoction	SAP of phlegm and blood stasis type	Improve myocardial ischemia and the high viscosity and hypercoagulable state of hemorheology	Xiong. (2014), Li and Muhati. (2016), Wang and Zhai. (2016), Zhou and Shu. (2017), Zhao. (2018)
	Shexiang baoxin pill	AP of qi and blood stasis type	Prevent myocardial injury	Yue (2014)
Add and subtract THSWD alone	—	SAP complicated with heart failure	Improve serology, hemorheology and cardiac function, reduce the level of blood lipids and alleviates inflammatory reaction, delay the progress of heart failure	Jiang. (2009), Wang et al. (2020)
	Astragalus membranaceus	UAP	Maintain the function of vascular endothelium, regulate blood lipids	Zheng et al. (2010)
	Ginseng	CHD-AP	Improve heart function and blood lipid status	Hunting et al. (2005)

### Application of Combined Chemicals

Clinical research has shown that THSWD combined with conventional chemical drugs can decrease the levels of serum TC, TG and LDL in patients with UAP (He et al., 2011), thus playing a role in reducing blood lipid and preventing AS. In chemical drugs combination therapy, modified THSWD combined with sodium ozagrel for injection (Jing and Wang, 2009; Renhua and Li, 2017), or Shuxuening (Wang and Wang, 2008), *Salvia miltiorrhiza* injection (Yu, 2012), Agkistrodon halys antithrombotic enzyme (Rui, 2002), which can effectively improve microcirculation and myocardial ischemia, alleviate the symptoms of AP and prevent the occurrence of myocardial infarction in patients with UAP, and there are no obvious adverse reactions and toxic and side effects. For patients with SAP with qi deficiency and blood stasis syndrome (Liu and Zhang, 2014; Dai and Wu, 2017; Zhang, 2018; Yang, 2019; Wu and Su, 2020), integrated traditional Chinese and Western medicine has a clear therapeutic effect, which can obviously improve the treatment efficiency and the quality of life of patients, reduce the level of blood lipid and the degree and frequency of AP attack, so as to accelerate the relief of clinical symptoms and improve cardiac function. Studies have shown that THSWD combined with Baoyuan Decoction and Metoprolol can treat patients with AP of qi deficiency and blood stasis syndrome (Wang and Wang, 2018), relieve symptoms and improve left ventricular systolic function (Wang et al., 2018b; Wang and Jiang, 2019). The mechanism may be related to the regulation of hemorheology and the levels of N-terminal pro-brain natriuretic peptide (NT-proBNP) (Xiao et al., 2016), serum troponin I and MCP-1. Furthermore, THSWD combined with diltiazem has outstanding clinical efficacy in the treatment of coronary spasm AP, and which can significantly improve the level of blood lipid indexes compared with the control group (Liu, 2015a).

Besides, the researchers believe that on the basis of routine use of chemical drugs for anti-angina pectoris, THSWD combined with Gualou Xiebai Banxia Decoction is used to treat chest arthralgia of phlegm and blood stasis type (Yang et al., 2014; Wang, 2016), and THSWD combined with Chaihu Shugan Powder is added to treat UAP of qi stagnation and blood stasis type (Yuan and Fan, 2019), which has significant clinical efficacy, thus further controlling the attack of AP and improving the quality of life, reflecting the concept of prevention and treatment of both symptoms and root causes of disease in TCM. The combined prescription may have the effects of dilating blood vessels, anti-inflammation, reducing blood lipids, anti-shock, regulating immune function and reducing blood viscosity (Li et al., 2008), so as to relieve AP and protect myocardium. For the prevention and treatment of acute coronary syndrome (ACS includes UAP and myocardial infarction), the curative effect of THSWD combined with Gualou Xiebai Banxia Decoction plus low molecular heparin calcium (Zhu et al., 2011), and THSWD combined with Sini Powder plus conventional chemical drugs is more significant than that of chemical drugs alone (Gao, 2014). Other studies have found that Ginseng plus THSWD combined with rosuvastatin (Haoli, 2018), or Shengmai Powder combined with THSWD and isosorbide mononitrate in preventing and treating AP (Yan et al., 2012), can not only improve myocardial blood supply and heart function, but also reduce blood lipid, thereby alleviating the damage of myocardial cells. In addition, on the basis of routine chemicals treatment, THSWD combined with Shixiao Powder can improve the ischemic electrocardiogram (ECG) performance of chest obstruction caused by blood stasis (Liu, 2015b; Yu, 2015), THSWD combined with Zhishi Xiebai Guizhi decoction can alleviate the clinical manifestations of myocardial ischemia in chronic CHD (Chen, 2018; Cheng, 2019), and only taking THSWD also can improve the TCM syndrome and the quality of life of patients (Wang and Fu, 2017; Chen and Xia, 2018; Dong, 2018; Liu, 2019b; Jia, 2020). The clinical use of atorvastatin alone in the treatment of CHD has poor efficacy and large side effects, while adding THSWD and Xiebai Banxia Decoction can avoid these adverse reactions (Wang, 2018).

The above research showed that the integrated traditional Chinese and western medicine therapy may be superior to the single chemicals therapy in improving the pathological changes and clinical manifestations of cardiovascular diseases related to myocardial injury to a certain extent (Yu, 2015), (Huang, 2012; Yu, 2015; Yang et al., 2019; Yang and Zhou, 2019), but the specific mechanism needs to be further clarified.

### Use of Combined Prescriptions

THSWD combined with Zhenwu decoction can reduce left ventricular end-diastolic and end-systolic diameter, plasma levels of brain natriuretic peptide (BNP) and matrix metalloproteinase-9 (MMP-9), increase left ventricular ejection fraction (LVEF) and tissue inhibitor of metalloproteinase-1 (TIMP-1) (Joung et al., 2003; Xiao and Chen, 2015; Xiao and Gao, 2017). It can improve the symptoms and signs of patients with chronic systolic heart failure (CSHF is the heart failure caused by SAP in ischemic heart disease) of yang deficiency and blood stasis, and suppresses the degradation of extracellular matrix (ECM) to delay the occurrence of ventricular remodeling. Among them, MMP-9 and TIMP-1 play an important role in the occurrence and development of ventricular remodeling and heart failure (Siwik et al., 2000). MMP-9 is a marker reflecting the degradation of myocardial ECM and ventricular remodeling (Martos et al., 2009). Under normal circumstances, TIMP-1 inhibits the activity of MMP-9 in a state of dynamic balance, and if unbalanced, ventricular remodeling will be aggravated (Chesler et al., 1999; Bradham et al., 2002; Fedak et al., 2004; Kassiri et al., 2005). In addition, clinical observation of CSHF with yang deficiency and blood stasis showed that Shenfu decoction combined with THSWD could significantly relieve symptoms such as palpitation, shortness of breath, wheezing, dyspnea and chest pain, increase LVEF and decrease the contents of NT-proBNP and BNP in plasma (Wang and Li, 2017).

Moreover, many researchers believe that THSWD combined with Baoyuan Decoction can significantly improve the clinical symptoms of patients with AP of qi-deficiency and blood-stasis type with higher safety (Song, 2010; Li and Zhang, 2018; Zhang, 2019). The pharmacological study of the combined prescription confirmed part of the action mechanism of Baoyuan decoction combined with THSWD (Li and Zhang, 2018), including coronary artery dilation, improvement of microcirculation, protection of damaged myocardium, enhancement of myocardial contractility, anti-platelet aggregation, inhibition of thrombosis and so on. In the treatment of SAP of phlegm and blood stasis type with THSWD combined with Gualou Xiebai Banxia decoction, several studies have shown that the combined prescription can improve myocardial ischemia and the high viscosity and hypercoagulable state of hemorheology (Xiong, 2014; Li et al., 2015; Li and Muhati, 2016; Wang and Zhai, 2016; Zhao, 2018), so as to protect cardiomyocytes from further injury. In addition, research has confirmed that THSWD combined with Shexiang Baoxin Pill has a certain prevention and therapeutic effect on AP of qi and blood stasis type (Yue, 2014).

In the clinical study of combined prescriptions, THSWD combined with other prescriptions has significant clinical effect in the treatment of cardiovascular disease, which can improve the TCM syndrome of patients and their quality of life. However, there are few cases in these clinical studies, and there is a lack of research and analysis of large clinical samples and standardization of syndrome types. Secondly, due to the limited observation time, there are few objective indicators selected in the study, so the inferences of results need to be further verified.

### Add and Subtract Taohong Siwu Decoction

Early studies showed that after GE’s THSWD was used for SAP, the pain and ECG were obviously improved. Although there was no statistically significant difference compared with the chemicals control group, the improvement of clinical symptoms of the treatment group was better than that in the control group (Jiang, 2009). Moreover, THSWD can improve the clinical efficacy of conventional chemical drugs in the treatment of SAP complicated with heart failure, and further improve the indexes of serology, hemorheology and cardiac function in patients (Wang et al., 2020). At the same time, it also lowers blood lipids and alleviates inflammatory reactions, thus delaying the progress of heart failure. THSWD combined with *Astragalus membranaceus* were used to enhance the efficacy of routine drugs in the treatment of UAP (Zheng et al., 2010), by maintaining the function of vascular endothelium, regulating blood lipids and reducing the levels of plasma ET and hs-CRP, to improve ECG and clinical manifestation of the patients. Research have shown that Ginseng combined with THSWD in the treatment of AP patients with CHD can further increase the curative effect, improve the heart function and blood lipid status of patients and higher safety (Chen et al., 2019). Although the use of THSWD alone can improve the prevention and treatment of the MI caused by cardiovascular disease, there are few clinical studies, and there are still many internal mechanisms that are not clear.

## Summary and Discussion

As one of the classic prescriptions for promoting blood circulation and removing blood stasis, THSWD has certain effects on the prevention and treatment of cardiovascular diseases (CHD, myocardial infarction, etc.). On the whole, it aims to control or delay the progression of CHD, alleviate the symptoms and frequency of myocardial ischemia and AP, thereby improving the quality of life, preventing myocardial infarction and prolonging life (Montalescot et al., 20132013).

At present, many studies have shown that THSWD can protect cardiomyocytes and improve cardiac function by inhibiting inflammatory reaction, antioxidant stress, inhibiting platelet aggregation, prolonging clotting time, anti-fibrosis, reducing blood lipids, anti-atherosclerosis, improving hemorheology and vascular lesions, regulating related signal pathways and so on. These possible mechanisms not only provide some research paths for researchers, but also provide clinicians with beneficial choices in the prevention and treatment of cardiovascular diseases, which is also a kind of welfare and hope for patients with cardiovascular diseases!

However, there are still some limitations and uncertainties in the research of prescriptions. According to the current research, THSWD contains many active ingredients (Wu, 2011; Li and Guo, 2016; Wang and Peng, 2017; WangChen and Han, 2019; Zhao and Liu, 2019; Nie and Cheng, 2020), including ligustilide, catalpol, paeoniflorin (Zhang et al., 2012; Zhou et al., 2013; Chen et al., 2015; Chen et al., 2018b; Qian et al., 2015; Zhai and Guo, 2016; Liu et al., 2019b; Liu et al., 2020), paeonional lactonine, amygdalin (Kung et al., 2021), kaempferol (Zhou et al., 2015; Feng et al., 2017; Chen et al., 2018a), quercetin (Ruiz et al., 2015; Roslan et al., 2017; Patel et al., 2018; Chen et al., 2020), paeonol, ferulic acid (Dai and Wu, 2017), benzoic acid, coumaric acid, caffeic acid, gallic acid, and hydroxysafflor yellow A (Song et al., 2005; Ji et al., 2008; Ji et al., 2009), etc., (Table 2). However, the molecular biological mechanism of which or several components play a role needs to be further studied and elucidated. Moreover, there are still many unknown ingredients in THSWD that have not been discovered and studied, and the pharmacological effects and mechanisms of these ingredients still need to be explored. In addition, although there are many clinical studies on the prevention and treatment of cardiovascular disease with THSWD, due to the blind sampling selection, short-term follow-up time and drop-out, the long-term effect of the study cannot be determined. Moreover, due to the lack of clear mechanisms of action, the combined use of THSWD with other prescriptions and chemical drugs is ambiguous and confusing to some extent, and there is still a lack of clear systematic evaluation of its efficacy and safety in the prevention and treatment of cardiovascular diseases. In addition, the dosage of each Chinese medicine component of THSWD in the literature research was inconsistent, and the quality of TCM was uneven, which may adversely affect the results of the study. Therefore, the results of the study may have a certain degree of psychological comfort tendency, which is not universal, and as the drug composition and dose are not standardized and quantified, the conclusion may have errors.

**TABLE 2:** The main active ingredients from THSWD in protection of cardiomyocytes.

**Chinese medicine**	**Ingredients**	**Pharmacological effects**	**References**
*Persicae semen*	β-sitosterol	Anti-oxidative stress	Wong et al. (2014), Koc et al. (2021)
*Carthami flos*			
*Angelicae sinensis radix*			
*Paeoniae radix alba*			
*Rehmanniae radix praeparata*			
*Chuanxiong rhizoma*	Tetramethylpyrazine (Ligustrazine)	Anti-atherosclerosis, anti-oxidation, anti-inflammation	Guo et al. (2016)
	Lactone component from *Ligusticum chuanxiong*	Regulate autophagy	Wang et al. (2018a)
*Persicae semen*	Amygdalin	Anti-inflammation, anti-oxidation	Kung et al. (2021)
*Carthami flos*	Hydroxysafflor yellow A	Protect vascular endothelium, Promote endothelial cell proliferation and angiogenesis	Ji et al. (2008), Ji et al. (2009)
	Baicalin	Cardiomyocytes protection, Macrophages polarization, lipid modulation	Liao et al., 2014, Yu et al. (2019), Xu et al. (2020)
	Quercetin	Anti-inflammation, anti-oxidation, mitochondrial function regulation, Cardiomyocytes protection	Ruiz et al. (2015), Roslan et al. (2017), Patel et al. (2018), Chen et al. (2020)
	Luteolin	Anti-inflammation, anti-oxidation, autophagy regulation	Luo et al. (2017), Li et al. (2019), Wu et al. (2020b)
	Kaempferol	Cardiomyocytes protection, inhibit inflammatory responses and oxidative stress	Zhou et al. (2015), Feng et al. (2017), Chen et al. (2018a)
Paeoniae radix alba	Paeoniflorin	improve ventricular remodeling, attenuate cardiac hypertrophy, anti-inflammation, anti-fibrosis, anti-oxidative stress	Zhang et al. (2012), Zhou et al. (2013), Chen et al. (2015), Chen et al. (2018b), Qian et al. (2015), Zhai and Guo. (2016), Liu et al. (2019a), Liu et al. (2019b), Liu et al. (2020), Wu et al. (2020a)

To sum up, THSWD has shown a broad prospect in the prevention and treatment of myocardial injury caused by cardiovascular diseases, but there are still many uncertainties. In the basic research of prescriptions and drugs, scientific research institutions should strengthen the quality control of drugs and further clarify the molecular biological mechanism of prevention and treatment of myocardial injury. In clinical research, clinical researchers should carry out multicenter, large sample prospective cohort studies to fully clarify its clinical efficacy and safety, providing sufficient and reliable theoretical and practical basis for the clinical application of THSWD in the prevention and treatment of myocardial injury. After thousands of years of traditional Chinese medicine practice challenges, the preservation of THSWD is inseparable from its practical value and historical significance. THSWD deserves further exploration by more researchers, so as to provide more potential utility for the prevention and treatment of other diseases besides myocardial injury, creating social value and ensuring people’s health, and realizing standardization, quantification and internationalization.
